# PKCδ activated by c-MET enhances infiltration of human glioblastoma cells through NOTCH2 signaling

**DOI:** 10.18632/oncotarget.6640

**Published:** 2015-12-17

**Authors:** Eunji Hwang, Ki-Chun Yoo, Seok-Gu Kang, Rae-Kwon Kim, Yan-Hong Cui, Hae-June Lee, Min-Jung Kim, Jae-Seong Lee, In-Gyu Kim, Yongjoon Suh, Su-Jae Lee

**Affiliations:** ^1^ Department of Life Science, Research Institute for Natural Sciences, Hanyang University, Seoul, Korea; ^2^ Department of Neurosurgery, Severance Hospital, Yonsei University College of Medicine, Seodaemun-gu, Korea; ^3^ Division of Radiation Effect, Korea Institute of Radiological and Medical Sciences, Seoul, Korea; ^4^ Laboratory of Radiation Exposure & Therapeutics, National Radiation Emergency Medical Center, Korea Institute of Radiological and Medical Sciences, Seoul, Korea; ^5^ Department of Biological Sciences, College of Science, Sungkyunkwan University, Seoul, Korea; ^6^ Department of Radiation Biology, Environmental Radiation Research Group, Korea Atomic Energy Research Institute, Daejeon, Korea

**Keywords:** PKCd, infiltration, glioblastoma, NOTCH2, c-MET

## Abstract

Poor prognosis of glioblastoma (GBM) is attributable to the propensity of tumor cells to infiltrate into the brain parenchyma. Protein kinase C (PKC) isozymes are highly expressed or aberrantly activated in GBM. However, how this signaling node translates to GBM cell invasiveness remains unknown. Here, we report that among PKC isoforms, PKCδ is strongly associated with infiltration of GBM cells. Notably, PKCδ enhanced Tyr418 phosphorylation of the non-receptor tyrosine kinase SRC, which in turn activated STAT3 and subsequent NOTCH2 signaling, ultimately leading to GBM cell invasiveness. Furthermore, we showed that PKCδ was aberrantly activated in GBM cells by c-MET, a receptor tyrosine kinase hyperactivated in GBM. In agreement, inhibition either component in the c-MET/PKCδ/SRC/STAT3 signaling axis effectively blocked the NOTCH2 signaling and invasiveness of GBM cells. Taken together, our findings shed a light on the signaling mechanisms behind the constitutive activation of PKCδ signaling in GBM.

## INTRODUCTION

Glioblastomas multiforme (GBM) is the most aggressive form of primary brain tumors with their tendency to invade surrounding healthy brain parenchyma, rendering them largely incurable. Despite advanced therapeutic strategies including surgery, radiotherapy and chemotherapy, the average survival time of a GBM patient is less than 16 months [1]. This poor prognosis is attributable to the rapid proliferation and aggressive infiltration of GBM cells. However, the mechanisms underlying GBM diffusion remain unclear. Identification of a causal molecular linkage might contribute to the development of targeted therapy leading to clinical outcome.

Protein kinase C (PKC) constitutes a family of serine/threonine kinases activated by a variety of external stimuli, such as hormones, growth factors and other membrane receptor ligands, that transduce intracellular signaling through phosphorylation of a large number of protein substrates[2]. Based on their structure and activation characteristics, the PKC family is classified into three subfamilies: classical or conventional PKC isozymes (PKCα, PKCβI, PKCβII and PKCγ), non-classical or novel PKC isozymes (PKCδ, PKCε, PKCη and PKCθ), and atypical PKC isozymes (PKCζ, PKCι and PKCλ)[3]. Given their many cellular roles and highly activation in cancers, not surprisingly a substantial body of evidence has linked PKC isozymes to carcinogenesis and cancer progression in many cancer types, including brain tumors [4–6]. However, since the availability of selective inhibitors of PKC isozymes has been limited, a particular role of specific PKC isozymes in GBM progression has still remained controversial, although specific tools such as siRNA (small interfering RNA) have allowed more detailed analysis of the function of this large family [2]. PKC isozymes have unique roles and differ in cellular functions in both normal and pathological conditions. In fact, the same PKC isozyme can have opposing roles in cancer, presumably due to complexity of their interactions with numerous substrates and the many secondary messenger systems coupled with their cellular and tissue-specific variability [2, 7].

In the present work, we demonstrate that among PKC isoforms, PKCδ is strongly associated with the invasiveness of GBM cells. Regarding the complexity of downstream signaling pathways, we sought to disentangle the network of PKCδ signaling components that are associated with GBM cell invasiveness. Importantly, we found that activation of PKCδ boosts the invasiveness of GBM cells by stimulating NOTCH2 signaling, which has been considered a novel therapeutic target for GBM treatment [8, 9]. PKCδ strengthened NOTCH2 signaling via SRC/STAT3 activation, ultimately leading to mesenchymal transformation. Moreover, we found that PKCδ is activated by a receptor tyrosine kinase c-MET, which has been found to be highly overexpressed in GBM patients and correlated with a median survival [10]. Collectively, our findings suggest that the c-MET/PKCδ drives to activation of SRC/STAT3/Notch2 signaling axis, implicating a novel therapeutic target to suppress the invasiveness of GBM.

## RESULTS

### PKCδ is a critical contributor to GBM infiltration

**Figure 1:**
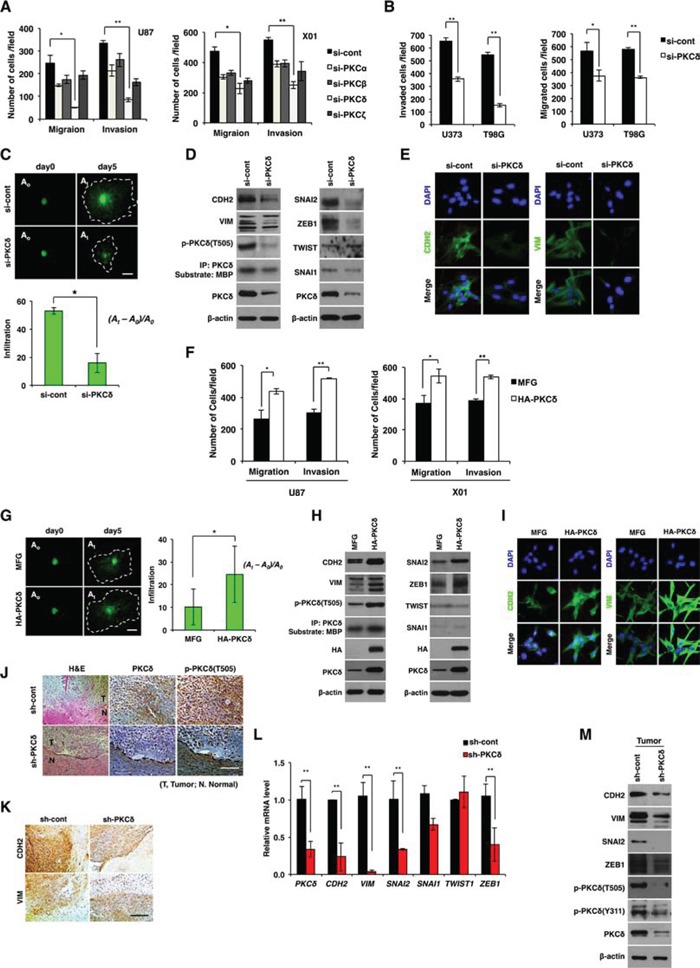
Effect of PKCδ on infiltration of GBM cells through mesenchymal transformation **A.** Migration and invasion assay in GBM cells transfected with control or PKC isoform siRNAs as indicated. **B.** Migration and invasion assay in GBM cells transfected with control or PKCδ siRNAs. **C.** Effect of PKCδ depletion on infiltration of U87 GBM cells in collagen-based matrix three-dimensional (3D) culture system. Scale bar, 100 μm. **D.** Western blot analysis for mesenchymal markers and regulators in U87 GBM cells transfected with control or PKCδ siRNAs. **E.** Immunocytochemistry for CDH2 and VIM in U87 GBM cells transfected with control or PKCδ siRNAs. **F.** Migration and invasion assay in GBM cells transduced with MFG or HA-PKCδ. **G.** Effect of PKCδ on infiltration of U87 GBM cells in collagen-based matrix 3D culture system. Scale bar, 100 μm. **H.** Western blot analysis for mesenchymal markers and regulators in U87 GBM cells transduced with MFG or HA-PKCδ. **I.** Immunocytochemistry for CDH2 and VIM in U87 GBM cells transduced with MFG or HA-PKCδ. **J, K.** Immunohistochemistry for p-PKCδ (J), and CDH2, VIM (K) in orthotopic U87 cell-xenograft tumors. U87 GBM cells were transduced with pSuper or PKCδ shRNA prior to injection to mice. Scale bar, 200 μm. **L, M.** q-RT PCR (L) and Western blot analysis (M) for mesenchymal markers and regulators in the orthotopic xenograft tumors. β-actin was used for a loading control. *, *P* < 0.05 versus control; **, *p*<0.01 versus control.

To determine the effect of PKC isozymes on the infiltration of GBM cells, we depleted individual PKC isoforms (PKCα, PKCβ, PKCζ, and PKCδ) by siRNA in the U87 GBM and patient-derived X01 GBM cells, and analyzed the migration and invasion of GBM cells in matrigel-coated (invasion assay) or uncoated (migration assay) transwells. Notably, knockdown of the isoform PKCδ was the most effective in suppressing the migration and invasion of both U87 and patient-derived GBM cells (Fig. 1A). Since migration and invasion in transwells could be underestimated by the relative decrease in the cell number caused by cell cycle arrest or death, we examined cell proliferation and cell death following treatment with PKCδ siRNA. Knockdown of PKCδ had no effect on cell proliferation or cell death (Fig. S1A, B), suggesting that migration and invasion assays in transwells were not affected by differential cell proliferation or cell death. To confirm the effect of PKCδ on invasiveness, we sought to extend our observations to additional GBM cell lines. Consistent with the above data, siRNA-mediated down-regulation of PKCδ also efficiently suppressed the migration and invasion of U373 and T98 GBM cells (Fig. 1B).

To further evaluate the role of PKCδ, we also examined the invasiveness of U87 GBM cells using 3D culture system in which collagen type I, a common ECM component, and Matrigel, with a composition similar to that of the basement membrane, were mixed and solidified in growth medium. For visualization, U87 GBM cells were transduced with green fluorescence protein (GFP) prior to the assay. Consistent with the above data, treatment with siRNA against PKCδ markedly decreased infiltration of U87 GBM cells compared to treatment with a control, scrambled siRNA, indicating that PKCδ is a critical regulator for GBM cell infiltration (Fig. 1C).

Since mesenchymal traits are correlated with GBM cell invasiveness [11, 12], we next examined whether PKCδ causes mesenchymal transformation of GBM cells. To this end, we analyzed the mesenchymal cell markers CDH2, VIM, and their regulators SNAI1, SNAI2, ZEB1 and TWIST1, after treatment with PKCδ siRNA. Depletion of PKCδ decreased CDH2 and VIM expression as well as that of their regulators SNAI2 and ZEB1 (Fig. 1D, 1E and Fig. S1C). In agreement with this result, siRNA-mediated knockdown of SNAI2 or ZEB1 suppressed the migratory and invasive properties of GBM cells (Fig. S1D). However, treatment with either siRNA against PKCα, -β or -ζ did not substantially decrease those mesenchymal markers, suggesting a distinctive role of PKCδ among the isoforms in mesenchymal transformation of GBM cells (Fig. S1E).

To further examine the direct involvement of PKCδ in the invasiveness of GBM cells, we next analyzed the effect of PKCδ overexpression on the migration and invasion of U87 GBM cells. As expected, and in contrast to knockdown, PKCδ overexpression enhanced the migratory and invasive properties of GBM cells (Fig. 1F, 1G). PKCδ overexpression also caused an increase in expression of the mesenchymal markers CDH2, VIM and their regulators SNAI2, ZEB1 in GBM cells (Fig. 1H, 1I and Fig. S1F).

To confirm the effect of PKCδ on the infiltration of GBM cells *in vivo*, we injected U87 GBM cells (5×10^4^ cells) orthotopically into the brains of athymic nude mice after transduction with PKCδ shRNA. 25 days later, histological staining of brain sections indicated that control shRNA-transduced U87 GBM cells formed tumors that had loosened and infiltrated into normal brain parenchyma, rendering the boundary between normal and tumor tissues indistinguishable (Fig. 1J). In contrast, tumors formed by U87 GBM cells transduced with PKCδ shRNA were relatively intact and dense, and appeared to exhibit a distinct margin. Consistent with *in vitro* data, shRNA-mediated knockdown of PKCδ *in vivo* decreased the levels of CDH2 and VIM as well as those of SNAI2, SNAI1, and ZEB1 in xenograft mice (Fig. 1K–1M). Taken together, these results suggest that PKCδ promotes infiltration of GBM cells through mesenchymal transformation.

### PKCδ promotes mesenchymal transformation through activation of SRC and STAT3

**Figure 2:**
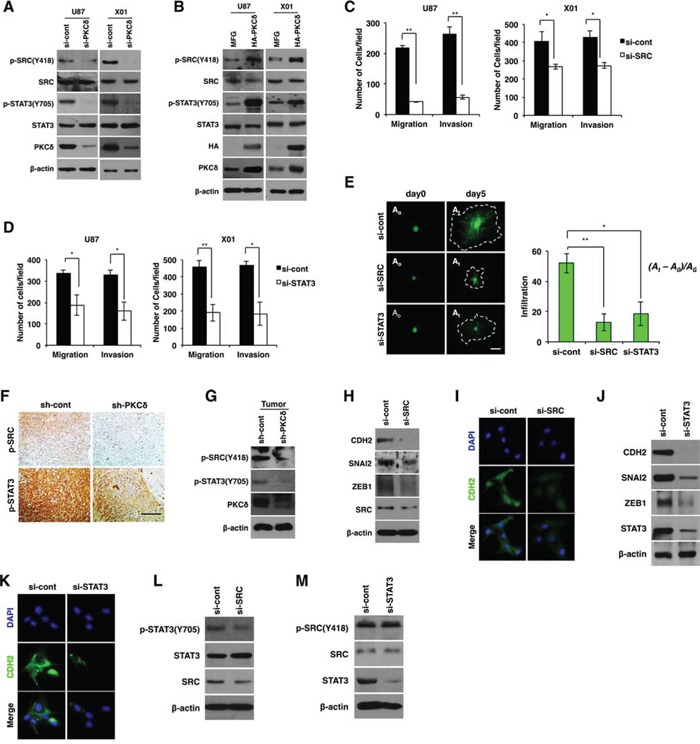
PKCδ promotes mesenchymal transformation through activation of SRC and STAT3 **A, B.** Western blot analysis for phosphorylation status of SRC and STAT3 in GBM cells transfected with control or PKCδ siRNAs (A), or transduced with MFG or HA-tagged PKCδ (B). **C, D.** Migration and invasion assay in GBM cells transfected with control or SRC (C), or STAT3 siRNAs (D). **E.** Infiltration of GBM cells transfected with control siRNAs or siRNAs against SRC or STAT3 in collagen-based matrix 3D culture system. Scale bar, 100 μm. **F, G.** Immunohistochemistry (F) and western blot analysis (G) for p-SRC and p-STAT3 in orthotopic U87 GBM cell-xenograft tumors. U87 GBM cells were transduced with control or PKCδ shRNA prior to orthotopic injection to mice. Scale bar, 200 μm. (H, I) Western blot analysis for CDH2, SNAI2 and ZEB1 **H.**, and immunocytochemistry for CDH2 **I.** in U87 GBM transfected with control or SRC siRNAs. (J, K) Western blot analysis for CDH2, SNAI2 and ZEB1 **J.**, and immunocytochemistry for CDH2 **K.** in U87 GBM cells transfected with control or STAT3 siRNAs. **L.** Western blot analysis for p-STAT3 in U87 GBM cells transfected with control or SRC siRNAs. **M.** Western blot analysis for p-SRC in U87 GBM cells transfected with control or STAT3 siRNAs. β-actin was used for a loading control. *, *P* < 0.05 versus control; **, *p*<0.01 versus control.

We next sought to determine which intracellular signaling route is activated by PKCδ that drives to infiltration of GBM cells. Since AKT, MAPK (mitogen-activated protein kinase), NF-κB (nuclear factor-kappaB), SRC and STAT (signal transducer and activator of transcription) signaling components are known to be downstream effectors of PKCδ, we analyzed the activation status of these signaling molecules after treatment with PKCδ siRNA. Notably, we found that PKCδ depletion caused a decrease in SRC Tyr418 phosphorylation and STAT3 Tyr705 and Ser727 phosphorylation, but did not alter activation of AKT, NF-κB or the MAPKs ERK (extracellular signal-regulated kinase), p38, or JNK (c-Jun N-terminal kinase) (Fig. 2A and Fig. S2A). Knockdown of either SRC or STAT3 did not activate PKCδ, indicating that PKCδ is upstream of SRC and STAT3 (Fig. S2B). To further confirm it, PKCδ was overexpressed in U87 GBM cell line and patient-derived X01 GBM cells and the activation status of SRC and STAT3 was analyzed. In agreement with these observations, the phosphorylation of SRC and STAT3 was enhanced by PKCδ overexpression (Fig. 2B).

Similar to the effect of PKCδ, depletion of SRC or STAT3 caused a decrease in migration and invasion of GBM cells in transwells (Fig. 2C, 2D). To confirm the effect, we also examined the invasiveness of GBM cells in 3D culture system after treatment with siRNA against SRC or STAT3. As expected, knockdown of either SRC or STAT3 inhibited the infiltrative properties of GBM cells in this system (Fig. 2E).

To validate the effects of PKCδ on SRC and STAT3 activation *in vivo*, we investigated the phosphorylation status of SRC and STAT3 by immunohistochemical analysis of tissue sections of GBM formed from orthotopically injected U87 GBM cells in mice. In line with our *in vitro* data, we observed that p-SRC and p-STAT3 were diminished in tumors formed by PKCδ-depleted GBM cells compared with tumors formed from scrambled shRNA-transduced GBM cells (Fig. 2F). Immunoblotting analyses conducted in parallel on the same tumor tissues confirmed the immunohistochemistry results (Fig. 2G).

Because our data indicated that PKCδ promoted mesenchymal transformation of GBM cells, we next examined whether inhibition of SRC and STAT3 also suppresses mesenchymal transformation. To this end, we analyzed CDH2, SNAI2, and ZEB1 after treatment of GBM cells with siRNA against SRC or STAT3. SRC depletion decreased CDH2 and VIM expression as well as that of their regulators SNAI2 and ZEB1 (Fig. 2H, 2I and Fig. S2C). Similarly, STAT3 depletion also caused a decrease in the levels of these mesenchymal signature proteins (Fig. 2J, 2K and Fig. S2C).

Since we found that SRC and STAT3 were activated by PKCδ, we next determined the sequence of PKCδ signal-activation events. Importantly, knockdown of SRC led to a decrease in the p-STAT3, whereas STAT3 depletion did not change the p-SRC level (Fig. 2L, 2M). To further confirm the SRC/STAT3 signaling axis, we treated GBM cells with SRC inhibitor PP2 and analyzed the phosphorylation status of STAT3. Consistently, inhibition of SRC activity attenuated STAT3 phosphorylation (Fig. S2D). These data indicate that PKCδ activates SRC, which in turn activates STAT3 to trigger the mesenchymal transformation underlying the infiltrative behavior of GBM cells.

### PKCδ/SRC/STAT3 signaling contributes to mesenchymal transformation via activation of NOTCH2

**Figure 3:**
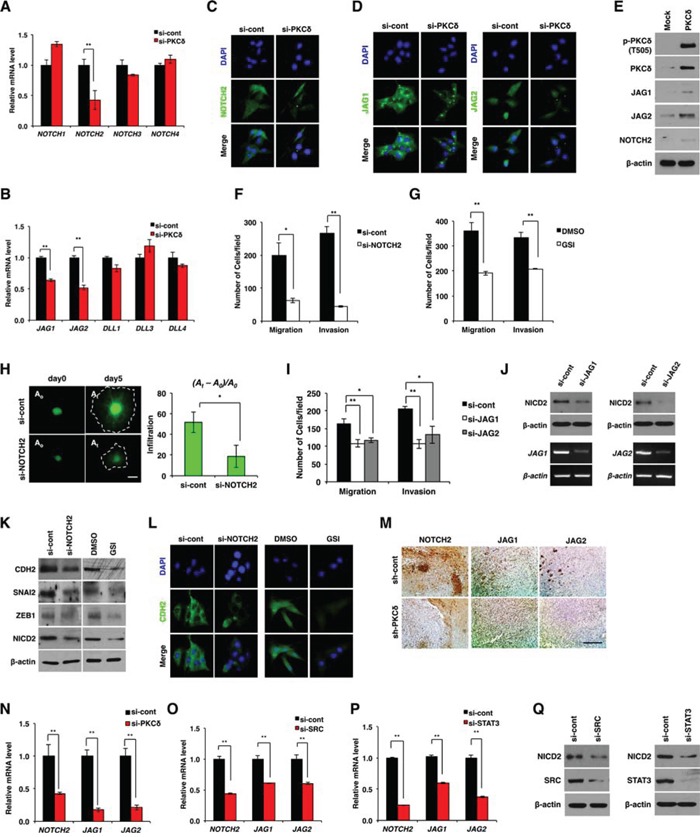
NOTCH2 is required for PKCδ-associated mesenchymal transformation **A, B.** qRT-PCR for NOTCH isoforms (A) and the ligands (B) in U87 GBM cells transfected with control or PKCδ siRNAs. **C, D.** Immunocytochemistry for NOTCH2 (C) and ligands JAG1 and -2 (D) in U87 GBM cells transfected with control or PKCδ siRNAs. **E.** Western blot analysis for NOTCH2 and its ligands JAG1 and -2 in U87 GBM cells transduced with MFG or HA-PKCδ. **F, G.** Migration and invasion assay in U87 GBM cells transfected with NOTCH2 siRNAs (F) or treated with γ-secretase inhibitor (GSI) (G), as compared to control. **H.** Infiltration of U87 GBM cells transfected with control or NOTCH2 siRNAs in collagen-based matrix 3D culture system. Scale bar, 100 μm. **I.** Migration and invasion assay in U87 GBM cells transfected with control siRNAs or siRNAs against JAG1 or -2. **J.** Western blot analysis for NICD2 in U87 GBM cells transfected with control siRNAs or siRNAs against JAG1 or -2. **K, L.** Western blot analysis for CDH2, SNAI2 and ZEB1 (K), and immunocytochemistry for CDH2 (L) in U87 GBM cells transfected with NOTCH2 siRNAs or treated with GSI. **M, N.** Immunohistochemical staining (M) and qRT-PCR (N) for NOTCH-2, JAG1 and -2 in orthotopic xenograft tumors formed by U87 GBM cells transduced with control (pSuper) or PKCδ shRNAs. Scale bar, 200 μm. **O, P.** qRT-PCR for NOTCH-2, JAG1 and -2 in U87 GBM cells transfected by control siRNAs or siRNAs against SRC (O) or STAT3 (P). **Q.** Western blot analysis for NICD2 in U87 GBM cells transfected by control siRNAs or siRNAs against SRC or STAT3. β-actin was used for a loading control. *, *P* < 0.05 versus control; **, *p*<0.01 versus control.

We next examined whether PKCδ is involved in NOTCH signaling, which has been found to play an important role in the pathogenesis of GBM [8, 9]. To this end, we examined the expression levels of NOTCH receptors and ligands. We found that siRNA-mediated PKCδ knockdown decreased levels of transcripts for the NOTCH2 receptor and its ligands JAG1 and -2 (Fig. 3A, 3B and Fig. S3A, B). Consistent with this, immunocytochemical analyses confirmed that PKCδ depletion decreased protein levels of NOTCH2 and its ligands JAG1 and -2 (Fig. 3C, 3D). In parallel with these findings, PKCδ overexpression increased NOTCH2 and its ligands JAG1 and -2 (Fig. 3E). However, NOTCH2 depletion did not alter PKCδ phosphorylation, indicating that NOTCH2 signaling acts as a downstream effector of PKCδ (Fig. S3C).

Extending these data, we next examined whether NOTCH2 is associated with infiltration of GBM cells. We found that siRNA-mediated knockdown of NOTCH2 suppressed the migration and invasion of GBM cells in a manner similar to that of PKCδ knockdown (Fig. 3F and Fig. S3D). Among NOTCH isoforms, NOTCH2 knockdown was the most effective in suppressing GBM cell migration and invasion (data not shown). Since NOTCH signaling is initiated by cytoplasmic cleavage of the NOTCH intracellular domain (NICD), we also analyzed migration and invasion following treatment with GSI, which inhibits cleavage of the NICD. Consistent with the effect of siRNA, inhibition of γ-secretase effectively suppressed the migratory and invasive properties of GBM cells (Fig. 3G). To validate the effect of NOTCH2, we also examined the invasiveness of GBM cells in the 3D culture system after treatment with NOTCH2 siRNA. As expected, NOTCH2 depletion markedly inhibited the infiltration of GBM cells (Fig. 3H). Treatment with siRNA against NOTCH ligands JAG1 or -2 also suppressed the migration and invasion of GBM cells (Fig. 3I), an effect that might be attributable to a decrease in the levels of NICD2 (Fig. 3J). Since PKCδ promoted infiltration of GBM cells through mesenchymal transformation, we next examined whether NOTCH2 is involved in mesenchymal transformation as a downstream effector of PKCδ. As was observed with PKCδ siRNA, treatment with NOTCH2 siRNA or with GSI caused a decrease in CDH2, SNAI2, and ZEB1 expression (Fig. 3K, 3L and Fig. S3E). We further confirmed the regulation of NOTCH2 by PKCδ in *in vivo* condition. By immunohistochemical staining we observed that NOTCH2, JAG1 and -2 were diminished in tumors formed by U87 GBM cells transduced with PKCδ shRNA compared with control tumors formed from scrambled shRNA-transduced U87 GBM cells (Fig. 3M). In agreement with this data, qRT-PCR analysis also revealed that depletion of PKCδ decreased the transcripts of NOTCH2, JAG1 and -2 in the orthotopic xenograft tumors (Fig. 3N).

We next examined whether NOTCH2 can be increased by SRC and STAT3, whose activities in turn are increased by PKCδ. Importantly, down-regulation of either SRC or STAT3 caused a decrease in the NOTCH2 receptor and its ligands JAG1 and -2 (Fig. 3O–3Q and Fig. S3F, G). Taken together, these results suggest that PKCδ promotes mesenchymal transformation through activation of the SRC/STAT3/NOTCH2 signaling axis.

### PKCδ is a downstream effector of c-MET in GBM

**Figure 4:**
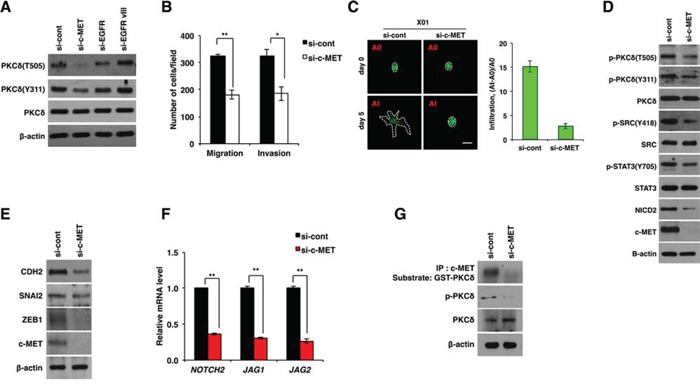
PKCδ is activated by c-MET **A.** Western blot analysis for activation status of PKCδ in U87 GBM cells transfected with control, c-MET, EGFR or EGFRvIII siRNAs. **B.** Migration and invasion assay in U87 GBM cells transfected with control or c-MET siRNAs. **C.** Infiltration of X01 GBM cells transfected with control or c-MET siRNAs in collagen-based matrix 3D culture system. Scale bar, 100 μm. **D, E.** Western blot analysis for activation status of PKCδ, SRC, STAT3 and NOTCH2 (D) or for CDH2, SNAI2 and ZEB1 (E) in U87 GBM cells transfected with control or c-MET siRNAs. **F.** qRT-PCR for NOTCH-2, JAG1 and -2 in U87 GBM cells transfected by control or c-MET siRNAs. **G.** Kinase assay of immunoprecipitated c-MET using GSC-PKCδ as a substrate and western blot analysis for p-PKCδ in U87 GBM cells transfected with control or c-MET siRNAs. β-actin was used for a loading control. *, *P* < 0.05 versus control; **, *p*<0.01 versus control.

c-MET and epidermal growth factor receptor (EGFR) are well-known tyrosine receptor kinases that are coexpressed in multiple cancers, including GBM [13, 14]. Since these tyrosine receptor kinases are closely associated with the malignant phenotypes of GBM, we examined whether PKCδ is an effector of these signaling pathways. Notably, siRNA-mediated knockdown of c-MET significantly decreased the phosphorylation of PKCδ, whereas depletion of EGFR or variant EGFRvIII had no such effect (Fig. 4A and Fig. S4A). In line with the data presented above, c-MET depletion attenuated the migration and invasion of GBM cells (Fig. 4B and Fig. S4B). Consistently, treatment with c-MET siRNA also mitigated the infiltration of patient-derived X01 GBM cells in 3-D spheroid (Fig. 4C).

Also in accord with these results, c-MET knockdown caused a decrease in the activation of SRC, STAT3 and NOTCH2, which are downstream effectors of PKCδ (Fig. 4D and Fig. S4C). In agreement, c-MET depletion caused a decrease in CDH2, SNAI2, and ZEB1 expression as observed in PKCδ or NOTCH2 knockdown (Fig. 4E and Fig. S4D). In addition, c-MET depletion decreased the transcript levels of NOTCH2, JAG1 and -2 (Fig. 4F and Fig. S4E). We next examined whether PKCδ is a direct substrate of c-MET kinase. Importantly, c-MET kinase assay revealed that c-MET could phosphorylate PKCδ, implicating PKCδ as a direct substrate of c-MET (Fig. 4G). To further confirm this finding, we also increased c-MET activation directly by treatment with hepatocyte growth factor (HGF), a well-known ligand for c-MET, and examined the changes in PKCδ phosphorylation and NOTCH2 levels. In agreement with the above data, we observed that PKCδ phosphorylation and NOTCH2 levels are proportionally increased in HGF concentration-dependent manner (Fig. S4F). Collectively, our results suggest that PKCδ is a downstream effector of c-MET and boosts infiltration of GBM cells through SRC/STAT3/NOTCH2 signaling axis.

**Figure 5:**
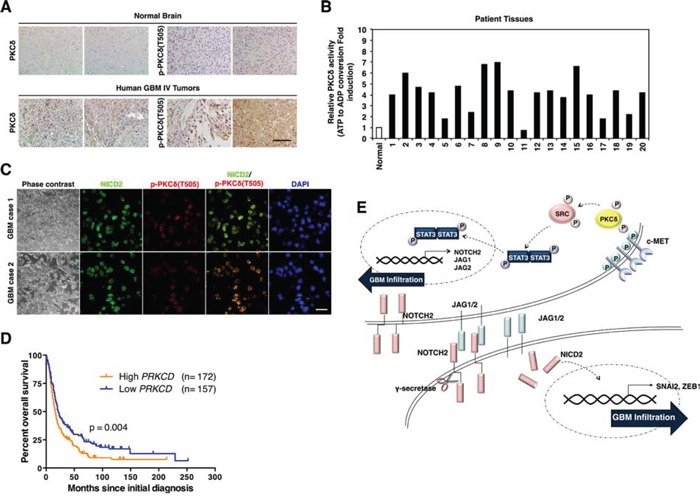
Clinical relevance of PKCδ in GBM patients **A.** Immunohistochemistry for PKCδ and p-PKCδ in human normal brain tissues and GBM patients (*n* = 30). Scale bar, 200 μm. **B.** PKCδ kinase activity in normal brain tissue and 20 cases of human GBM patients. **C.** Immunohistochemistry for co-staining of PKCδ and NICD2 in human GBM. Scale bar, 100 μm. **D.** Kaplan-Meier survival curves of high and low levels of PKCδ in human brain tumor patients with REMBRANDT database. **E.** Schematic model illustrating the PKCδ-associated signaling pathways leading to infiltration of GBM.

To evaluate the potential relevance of PKCδ as a clinical target, we next compared the levels of PKCδ in tissues of human GBM with that in normal brain counterparts. Importantly, immunohistochemical analyses revealed that PKCδ is highly expressed in GBM compared to normal tissues (Fig. 5A). The phosphorylation status was also higher in GBM than in normal tissue. To further confirm this, we compared PKCδ activity in 20 cases GBM patient tissues with that in non-neoplastic brain tissues. The results obtained were in agreement, showing that PKCδ activities were higher in most GBM than in non-neoplastic tissues (Fig. 5B). Since we found that PKCδ contributes to the infiltration of GBM cells through NOTCH2 signaling, we also examined the p-PKCδ and NOTCH2 levels in patient GBM tissues. By immunohistochemistry, we found that the p-PKCδ is co-localized with NICD2 in almost cells of GBM patient tissues (Fig. 5C). Since PKCδ boosts infiltration of GBM cells *in vitro* and is highly activated in human GBM tissues, we next evaluated whether PKCδ levels in human brain tumor correlated with patient survival. Evaluation of data in REMBRANDT database revealed that expression levels of PKCδ in human brain tumors are inversely correlated with the patient survival (Fig. 5D). In contrast, however, expression levels of other PKC isoforms (PKCα and -β) were proportional to the patient survival time, emphasizing the differential role of PKCδ from the isoforms (Fig S5). Although these retrospective data cannot determine whether PKCδ is an independent predictor of survival, these data suggest that PKCδ is a negative prognostic factor for human glioma patients. Taken altogether, these results suggest that PKCδ likely contributes to the infiltration of GBM cells *in vivo* through the c-MET/PKCδ/SRC/STAT3/NOTCH2 signaling axis as it does *in vitro* (Fig. 5E).

## DISCUSSION

Despite recent advances in anticancer therapeutic strategies, including surgery, chemotherapy and radiotherapy, the clinical outcomes for GBM patients have not shown a proportional improvement. This dismal prognosis is attributable to GBM invasiveness. Thus, identifying the pivotal molecular mediators of GBM infiltration is a prerequisite for developing targeted therapies to improve clinical outcomes of GBM patients.

In this study, we found that among PKC isoforms, PKCδ contributes to GBM cell invasiveness. Notably, PKCδ knockdown effectively suppressed migration and invasion of GBM cells, whereas PKCδ overexpression enhanced infiltration. Moreover, PKCδ promoted invasiveness of GBM cells through mesenchymal transformation. Since down-regulation of PKCδ has been associated with tumor promotion, it has been previously thought that PKCδ functions as a tumor suppressor [15–17]. However, in keeping with our observation, many recent studies have also reported that PKCδ contributes to tumor progression [18, 19]. These contrasting observations highlight the complexity of PKCδ signaling pathways and suggest that the different cellular responses could be caused by activation of different sets of downstream effectors. Following this notion, we sought to identify PKCδ downstream effectors that are associated with GBM cell infiltration. Importantly, we found that PKCδ increased phosphorylation of SRC, which in turn activated STAT3 and, ultimately, NOTCH2 signaling, leading to mesenchymal transformation.

Previous studies have strongly implicated the non-receptor tyrosine kinase SRC in the development, maintenance, progression, and invasiveness of several human cancers, including brain cancers [20]. Despite the finding that the SRC gene is not amplified or mutated, the activity of the corresponding protein was found to be higher in GBM compared to normal brain tissues. Moreover, the activation of SRC is known to be closely associated with poor prognosis of GBM [21, 22]. Similarly, no genetic alterations leading to STAT3 overexpression have been identified. However, approximately 70% of human cancers display persistent STAT3 activation, causing induction of oncogenes [23–25]. In keeping with this notion, our finding that STAT3 can be activated by PKCδ could explain the aberrant activation of STAT3 in GBM.

Our study also showed that PKCδ-enhanced STAT3 activation causes an increase in NOTCH2 signaling. By previous studies, NOTCH signaling has correlation with GBM progression [8, 26]. In particular, Chen *et al*. suggested that NOTCH2 is the predominant NOTCH receptor that contributes to the growth of GBM [27]. Consistent with these previous studies, we found that PKCδ increases expression of the NOTCH2 receptor and its ligands JAG1 and -2 through activation of SRC/STAT3. Collectively, our findings suggest that PKCδ is responsible for the persistent activation of NOTCH2 as well as SRC and STAT3, thereby contributing to GBM progression.

The tyrosine kinase c-MET has been reported to promote tumor formation and resistance to DNA damage, and expand the GBM stem-like cell population. Accordingly, numerous small molecule inhibitors targeting this pathway are at advanced stages of clinical development [28–30]. Notable in this context, we found that c-MET increases the activation of PKCδ. In agreement with this finding, c-MET inhibition decreased the activation of SRC and STAT3 and the expression of NOTCH2 receptor and its ligands JAG1 and -2, an effect similar to PKCδ depletion.

In summary, we have identified PKCδ downstream effectors that are associated with the GBM cell invasiveness. Importantly, PKCδ promoted mesenchymal transformation of GBM cells through activation of SRC/STAT3/NOTCH2 signaling. We also showed that PKCδ is activated by c-MET, linking the PKCδ signaling node to c-MET, a target of therapeutic agents at an advanced stage of clinical development. Collectively, our findings implicate the c-MET/PKCδ/SRC/STAT3/NOTCH2 signaling axis as a potential therapeutic target in GBM treatment.

## MATERIALS AND METHODS

### Cell culture

U87, U373 and T98G GBM cells were obtained from the Korean Cell Line Bank (KCLB, Seoul, Korea). The patient-derived X01 GBM cells were established from acutely resected human tumor tissues obtained with written informed consent from a 68-year-old woman with GBM. U87, U373, and X01 GBM cells were cultured in Dulbecco's modified Eagle Medium (Gibco, Korea, Seoul) and T98G cells were cultured in Minimum Essential Medium (Gibco), containing 1% penicillin and streptomycin, supplemented with 10% heat-inactivated fetal bovine serum. For visualization, U87 GBM cells were labeled by transduction with MSCV-GFP that was kindly provided from Hee-Yong Chung (Department of Medicine, Hanyang University).

### Chemical reagents and antibodies

Polyclonal antibodies to PKCδ, vimentin (VIM), TWIST11, SNAI1 (also known as SNAI1), SNAI2 (also known as SLUG), STAT3, SRC, IκB, NFκB, Jagged-1 (JAG1), Jagged-2 (JAG2) and CDH2 (also known as N-cadherin) were purchased from Santa Cruz (Santa Cruz, CA, USA). Polyclonal antibodies to p-PKCδ (Y311), phospho-STAT3 (Y705), AKT, p-AKT (S473), p-AKT (T308), ERK1/2, p-ERK1/2 (T202/204), Cleaved NOTCH1, JNK, p-P38, and monoclonal antibodies to HA, NOTCH3 and NOTCH4 were purchased from Cell Signaling Technology (Beverly, MA, USA). Polyclonal antibodies to phospho- PKCδ (T505), activated NOTCH2 and p-SRC(Y418) were purchased from Abcam and polyclonal antibody to ZEB1 was purchased from Sigma. Monoclonal antibody to p-JNK was purchased from BD. Anti-Human NOTCH-2 Intracellular Domain Antigen Affinity-purified Polyclonal Antibody was purchased from R&D systems. γ-secretase inhibitor (GSI) was purchased from Calbiochem (San Diego, CA, USA).

### Western blot analysis

Cell lysates were prepared by extracting proteins with lysis buffer [40 mM Tris–HCl (pH 8.0), 120 mM NaCl, 0.1% Nonidet-P40] supplemented with protease inhibitors. Proteins were separated by SDS-PAGE, and transferred to a nitrocellulose membrane (Amersham, Arlington Heights, IL, USA). The membrane was blocked with 5% non-fat dry milk in Tris-buffered saline, and incubated with primary antibodies overnight at 4°C. Blots were developed with a peroxidase-conjugated secondary antibody, and proteins visualized by enhanced chemiluminescence (ECL) procedures (Amersham), using the manufacturer's protocol.

### Invasion and migration assays

For invasion assay, the cells (2 × 10^4^ cells/well) were loaded in the upper well of the transwell chamber (8-μm pore size; Corning Glass, Seoul, Korea) that was precoated with 10 mg/ml growth factor-reduced matrigel (BD Biosciences, Seoul, Korea) on the upper side of the chamber, with the lower well filled with 0.8 ml of DMEM. After incubation for 48 h at 37°C, non-migrated cells on the upper surface of the filter were removed with a cotton swab, and migrated cells on the lower surface of the filter were fixed and stained with a Diff-Quick kit and photographed. Invasiveness was determined by counting cells in five microscopic fields per well, and the extent of invasion was expressed as an average number of cells per microscopic field. Cells were imaged by phase contrast microscopy (Leica Microsystems, Bannockburn, IL, USA). For migration assay, we performed the same procedure using the same chambers with control inserts that contained the same type of membrane but without the Matrigel coating. Infiltration of GBM cells was also analyzed in 3D spheroid cultures. Collagen type I (2 mg/ml) and matrigel (11%) were deposited in individual chamber slides and polymerized in growth medium. GFP-expressing U87 GBM spheroid was then seeded on the collagen-based matrix and the collagen-based matrix was covered on it. After 48 h, infiltration of GBM cells was analyzed under microscope.

### Immunocytochemistry

Cells were fixed with 4% paraformaldehyde and permeabilized with 0.1% Triton X-100 in PBS. Following cell fixation, cells were incubated with the appropriate primary antibodies in a solution of PBS with 1% bovine serum albumin and 0.1% Triton X-100 at 4°C overnight. Antibodies used were as follows: -CDH2 (rabbit polyclonal antibody, 1:200), -VIM (rabbit polyclonal antibody, 1:200), -activated NOTCH2 (rabbit polyclonal antibody, 1:200), -JAG1 (rabbit monoclonal antibody, 1:200), and -JAG2; rabbit polyclonal antibody, 1:200). Cells were visualized using anti-rabbit or anti-mouse Alexa Flour 488 (Molecular Probes). Nuclei were counterstained using 4,6-diamidino-2-phenylindole (DAPI; Sigma, St Louis, MO, USA). Stained cells were visualized with a fluorescencemicroscope (Olympus IX71, Seoul, Korea).

### Transfection

Small interfering RNA (siRNAs) or DNA plasmids were introduced into cells using a Microporator-mini (Digital Bio-Technology, Seoul, Korea) according to the procedure recommended by the manufacturer. Cells were harvested at 48 h for subsequent experiments. All siRNAs were purchased from Genolution Pharmaceuticals (Seoul, Korea). A validated shRNA (Mbiotech, Seoul, Korea) was cloned into pSuper vector.

### Transduction

The full length of PKCδ was cloned into retroviral vector pMFG. For viral production, 293T cells were transfected with retroviral vector pMFG or pMFG-HA-PKCδ, or pMSCV-GFP using the lipofectamine2000 (Invitrogen, Carlsbad, CA, USA). 48 h after the transfection, viral supernatant was collected and passed through a 0.45 μm filter, and the viral supernatant was then used for transduction with supplementation of 8 μg/ml polybrene (Sigma, St. Louis, MO, USA).

### Reverse transcription PCR

Total RNA was isolated manually using the Trizol (Invitrogen Carlsbad, CA, USA). All qRT-PCR was performed using KAPA SYBR FAST qPCR kit from KAPA Biosystems (Wilmington, MA, USA), according to the manufacturer's recommendations. Reactions were carried out in Rotor Gene Q (Qiagene, Seoul, Korea) and results were expressed as fold change calculated by the ΔΔCt method relative to the control sample. *GAPDH* was used as an internal normalization control.

### Immune complex kinase assay

Proteins in cell lysates were immunoprecipitated with primary antibody at 4°C for 4 h. The immunoprecipitates were washed with kinase reaction buffer (50 mM HEPES, pH 7.5, 10 mM MgCl_2_, 1mM dithiothreitol, 2.5 mM EGTA, 1 mM NaF, 0.1 mM Na_3_VO_4_ and 10 mM glycerophosphate) and then resuspended in 20 μl of kinase reaction buffer. The kinase assay was initiated by adding 20 μl of kinase reaction buffer, containing 10 μg of substrate and 2 μCi of [γ-^32^P] ATP (Valent Pharmaceuticals International, Laval, QC, Canada). The reactions were carried out at 30°C for 30 minutes and terminated by adding SDS sample buffer. The reaction products were analyzed by SDS-PAGE and autoradiography. Also, the activity of PKCδ was measured by PKC kinase activity kit (Enzo Life Science, Seoul, Korea). Human GBM patient tissues were kindly provided by Prof. Kang in Severance Hospital. Prior informed consents were obtained from the GBM sample donors.

### Animal experiments

X01 GBM cells (5 × 10^4^ cells/mouse) were orthotopically injected into athymic BALB/c nude mice (6 weeks old, KBT Oriental, Charles River Grade, Tosu, Saga, Japan) through the burr hole in to the right striatum at the coordinates of 2.0 mm lateral to the midline, 3.0 mm in vertical and anterior-posterior at the zero point to the bregma (*n* = 5/group). Mice were sacrificed around 3.5 weeks and the mouse brains were sectioned at the center of burr hole and tumor sizes were analyzed following hematoxylin and eosin staining. Mice were housed in microisolator cages under sterile conditions and lighting, temperature, and humidity were controlled centrally. This study was approved by the Institutional Animal Care and Use Committee of Yonsei University.

### Immunohistochemistry

Mice were sacrificed and tumor tissues were fixed in formalin for the preparation of paraffin sections. Paraffin-embedded tissue sections were deparaffinized in xylene, 95, 90 and 70% ethanol, followed by phosphate-buffered saline (PBS). Epitopes were unmasked with 20 mg/ml proteinase K in PBS with 0.1% Triton X-100. Sections were stained with hematoxylin and eosin or immunostained with the PKCδ, p-PKCδ, VIM, CDH2, p-SRC, p-STAT3, NOTCH2, JAG1, JAG2 antibodies. After washing in PBS, 1:200 dilution of biotinylated goat anti-rabbit IgG or anti-mouse IgG antibody in blocking solution was applied to the sections. After washing in PBS, ABC reagent was applied to the sections. Color reaction was performed with 3,3′ diaminobenzidine (Vector Laboratories, Burlingame, CA, USA) and slides were washed with PBS. After counterstaining with hematoxylin and clearing with graded ethanol series and xylene, the sections were mounted with Canada balsam. Observation and photography were conducted by IX71 microscope (Olympus, Seoul, Korea).

### Tissue array

The levels of PKCδ, p-PKCδ and NOTCH2 were analyzed using tissue array containing 30 human GBMs and 2 normal brain tissues (ISU ABXIS, Seoul, Korea).

### Kaplan-meier survival analysis

The National Cancer Institute's Repository for Molecular Brain Neoplasia Data (REMBRANDT, http://www.betastasis.com/glioma/rembrandt, accessed June 2015) was evaluated for correlations between clinical outcome/survival and *PRKCD* gene expression in human brain tumor biopsies. For REMBRANDT, “high expression” is defined as upper than threshold 7.4; “low expression” is defined as lower than the threshold. Similarly, patient survival curves were also obtained for other isoforms *PRKCA* and *PRKCB*.

### Statistical analysis

All experimental data are reported as means and the error bars represent the experimental standard errors. Statistical analyses were performed using parametric Student t-tests.

## SUPPLEMENTARY FIGURES


