# Supplementary material for: PKCδ activated by c-MET enhances infiltration of human glioblastoma cells through NOTCH2 signaling
Source: Oncotarget. 2015 Dec 17;7(4):4890–902. doi: 10.18632/oncotarget.6640 (PMC4826251; doi:10.18632/oncotarget.6640)
Supplement: Supplementary file 1 [file oncotarget-07-4890-s001.pdf]

SUPPLEMENTARY FIGURES

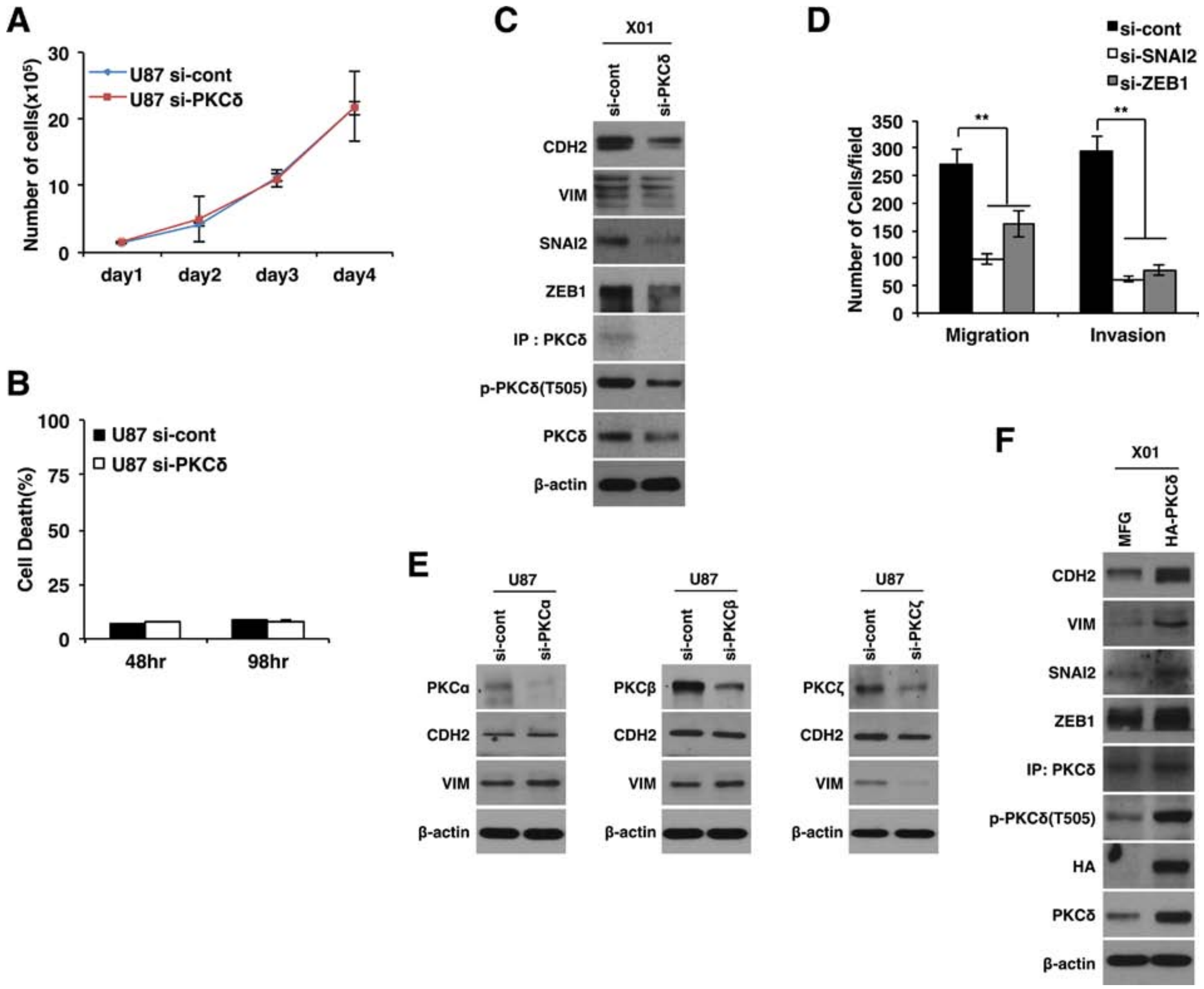

Supplementary Figure S1: Effect of PKCδ on infiltration of GBM cells through mesenchymal transformation. A. Cell growth curves of U87 GBM cells transfected with control or PKCδ siRNAs. **B.** Measurement of cell death in U87 GBM cells transfected with control or PKCδ siRNAs by FACS analysis after staining with propidium iodide. **C.** Western blot analysis for mesenchymal cell markers (CDH2, VIM) and regulators (SNAI1, ZEB1, TWIST1 and SNAI2) in X01 GBM cells transfected with control or PKCδ siRNAs. **D.** Migration and invasion assay in U87 GBM cells transfected with control, SNAI2 or ZEB1 siRNAs. **E.** Western blot analysis for mesenchymal cell markers (CDH2, VIM) in U87 GBM cells transfected with siRNA against PKCα, -β, -ζ or scrambled negative control siRNA. **F.** Western blot analysis for CDH2, VIM, SNAI2 and ZEB1 in X01 GBM cells transduced with empty vector MFG or HA-PKCδ. β-actin was used for a loading control. **, $p<0.01$ versus control.

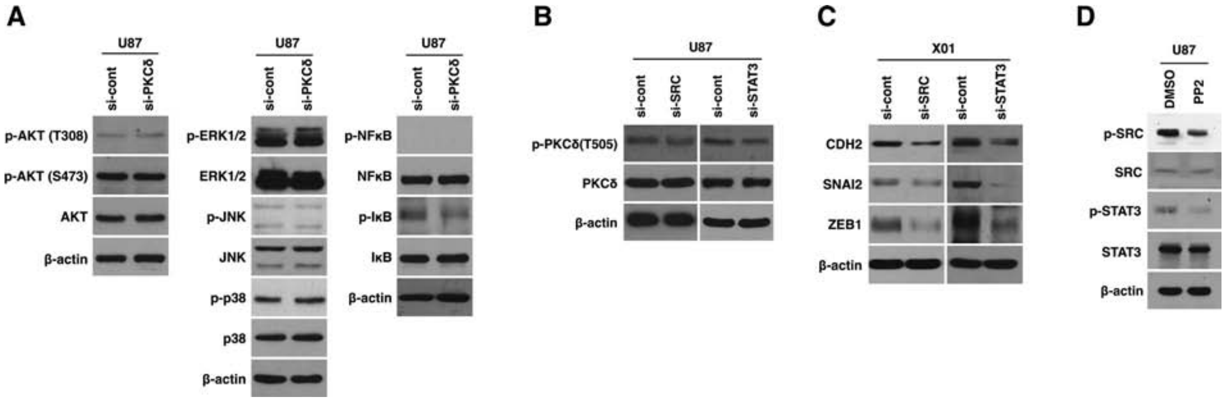

Supplementary Figure S2: Downstream effectors of PKCδ on infiltration of GBM cells through mesenchymal transformation. A. Western blot analysis for activation status of AKT, MAPK (ERK, p38, JNK) and NFκB in U87 GBM cells transfected with control or PKCδ siRNAs. **B.** Western blot analysis for activation status of PKCδ in U87 GBM cells transfected with control or PKCδ siRNAs. **C.** Western blot analysis for CDH2, SNAI2 and ZEB1 in X01 GBM cells transfected with control siRNAs or siRNAs against SRC or STAT3. **D.** Western blot analysis for phosphorylation status of STAT3 after treatment with SRC inhibitor PP2. β-actin was used for a loading control.

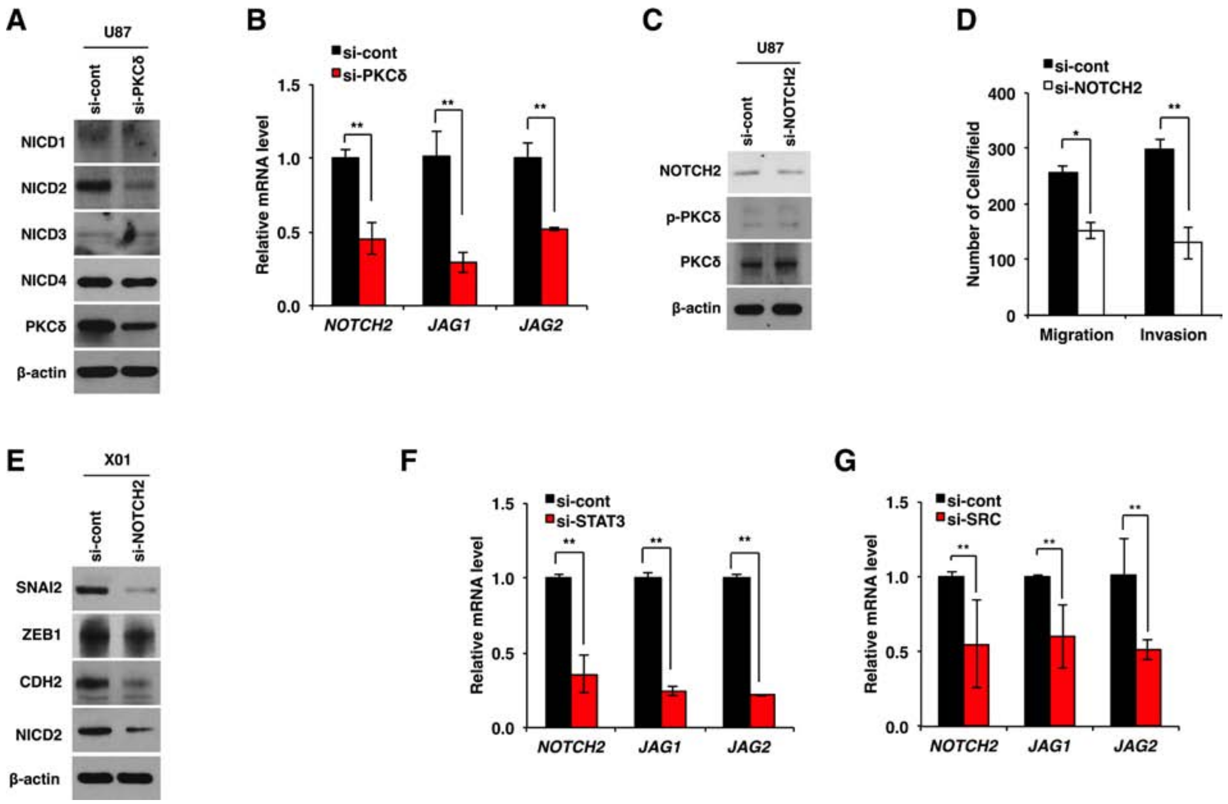

Supplementary Figure S3: PKCδ boosts mesenchymal transformation through NOTCH2. **A.** Western blot analysis for NOTCH intracellular domain (NICD)-1, -2, -3, and -4 in U87 GBM cells transfected with control or PKCδ siRNAs. **B.** qRT-PCR for NOTCH2, JAG1 and JAG2 in X01 GBM cells transfected with control or PKCδ siRNAs. **C.** Western blot analysis for phosphorylation status of STAT3 in U87 GBM cells transfected with control or NOTCH2 siRNAs. **D.** Migration and invasion assay in X01 GBM cells transfected with control or NOTCH2 siRNAs. **E.** Western blot analysis for CDH2, SNAI2 and ZEB1 in X01 GBM cells transfected with control or NOTCH2 siRNAs. **F, G.** qRT-PCR for NOTCH2, JAG1 and JAG2 in X01 GBM cells transfected with control siRNAs or siRNAs against STAT3 (F) or SRC (G). β-actin was used for a loading control. *, $P < 0.05$ versus control; **, $p<0.01$ versus control.

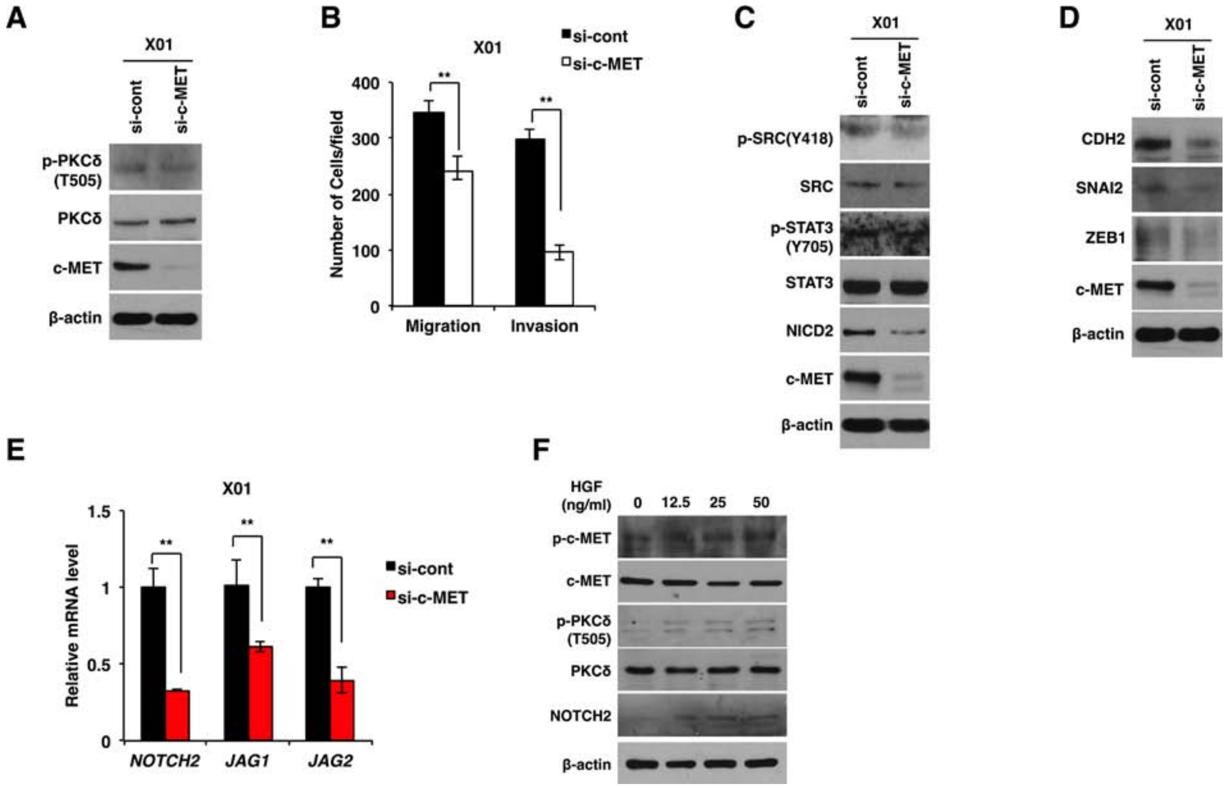

Supplementary Figure S4: PKCδ is activated by c-MET in GBM. A. Western blot analysis for activation status of PKCδ in X01 GBM cells transfected with control or c-MET. **B.** Migration and invasion assay in X01 GBM cells transfected with control or c-MET siRNAs. **C.** Western blot analysis for activation status of SRC, STAT3 and NOTCH2 in X01 GBM cells transfected with control or c-MET siRNAs. **D.** Western blot analysis for CDH2, SNAI2 and ZEB1 in X01 GBM cells transfected with control or c-MET siRNAs. **E.** qRT-PCR for NOTCH-2, JAG1 and -2 in X01 GBM cells transfected by control or c-MET siRNAs. **F.** Western blot analysis for phosphorylation status of PKCδ and NOTCH2 levels after treatment with HGF. β-actin was used for a loading control. **, p<0.01 versus control.

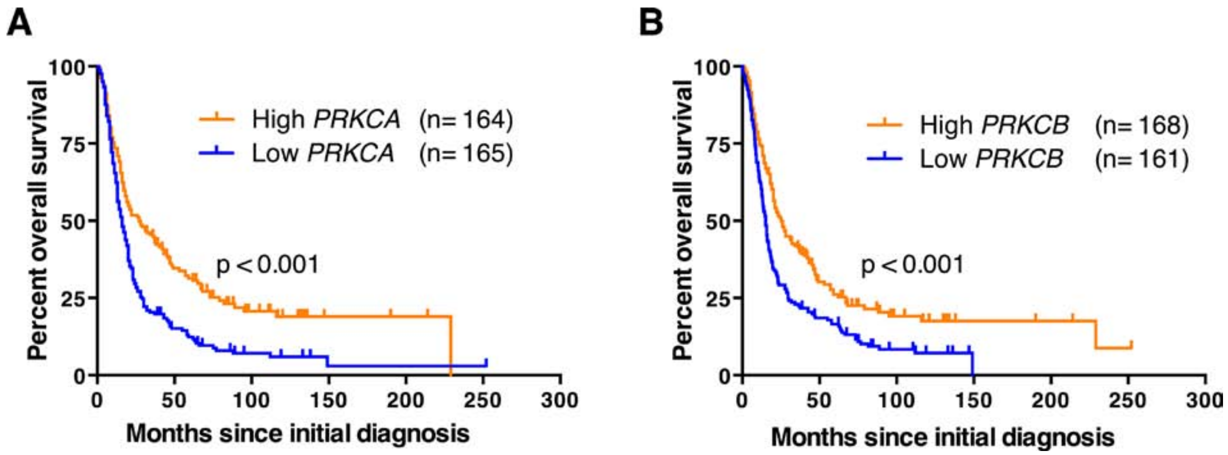

Supplementary Figure S5: Kaplan-Meier survival curves of human brain tumor patients expressing high and low levels of *PRKCA* A. and *PRKCB* B. in REMBRANDT database.
